# Outcomes of Older Patients with Resectable Colorectal Liver Metastases Cancer (CRLM): Single Center Experience

**DOI:** 10.3390/curroncol28030176

**Published:** 2021-05-18

**Authors:** Rami Nassabein, Laura Mansour, Corentin Richard, Franck Vandenbroucke-Menu, Francine Aubin, Jean-Pierre Ayoub, Michel Dagenais, Real Lapointe, Richard Letourneau, Marylène Plasse, André Roy, Simon Turcotte, Mustapha Tehfe

**Affiliations:** 1 Department of Medicine, Hematology-Oncology Division, Centre Hospitalier de l’Université de Montréal (CHUM), Montréal, QC H2X 0C1, Canada; rami.nassabein@hotmail.com (R.N.); lora-rm@hotmail.com (L.M.); francine.aubin.med@ssss.gouv.qc.ca (F.A.); jean-pierre.ayoub.chum@ssss.gouv.qc.ca (J.-P.A.); 2 Centre de Recherche du Centre Hospitalier de l’Université de Montréal (CRCHUM), Montréal, QC H2X 0A9, Canada; corentin.richard@umontreal.ca; 3 Department of Surgery, Hepatopancreatobiliary Division, Centre Hospitalier de l’Université de Montréal (CHUM), Montréal, QC H2X 0C1, Canada; franck.vandenbroucke@umontreal.ca (F.V.-M.); michel.dagenais.chum@ssss.gouv.qc.ca (M.D.); real.lapointe@umontreal.ca (R.L.); richard.letourneau@umontreal.ca (R.L.); marylene.plasse.med@ssss.gouv.qc.ca (M.P.); andre.roy.med@ssss.gouv.qc.ca (A.R.); simon.turcotte.med@ssss.gouv.qc.ca (S.T.)

**Keywords:** colon cancer, CRLM, hepatic metastectomy, perioperative chemotherapy, NLR, comprehensive geriatric assessment

## Abstract

Surgery is the only potential curative option of CRLM if resectable. The curative approach in patients over 70 years old is challenging mainly because of comorbidities and other geriatric syndromes. Herein, we report outcomes of older patients with resectable CRLM in our center. We retrospectively analyzed characteristics and outcomes of older patients with CRLM operated at “Centre Hospitalier de l’Université de Montréal” (CHUM) between 2010 and 2019. We identified 210 patients aged ≥70 years with a median age of 76 (range: 70–85). CRLM were synchronous in 56% of patients. Median disease-free survival (DFS) was 41.3 months. Median overall survival (OS) was 62.2 months and estimated 5-year survival rate was 51.5% similar to those of younger counterparts. Patients with metachronous CRLM had a trend to a higher OS compared to those with synchronous disease (67.2 vs. 58.7 months; *p* = 0.42). Factors associated with lower survival in the multivariate analysis were right-sided tumors and increased Charlson Comorbidity index (CCI). Survival outcomes of patients aged ≥70 years were comparable to those of younger patients and those reported in the literature. Age should not be a limiting factor in the curative management of older patients with resectable CRLM.

## 1. Introduction

Colorectal cancer is the fourth most common cancer worldwide and represents about 6% of all new cancer cases [1]. In Canada, colorectal cancer is the third leading cause of cancer deaths with a five-year survival rate of 65% [2]. Age remains an important risk factor, as incidence of colorectal cancers increase in patients more than 70 years old [3]. With the increasing life span of the population, the number of older patients with CRLM is rising, leading to required optimal management [4].

Surgery remains the only potentially curative treatment for localized disease or for tumors with limited liver metastases [5,6]. Adjuvant chemotherapy in Stage II is usually restricted to patients with high risk, although controversial, and is widely recommended for stage III disease. 5-fluorouracil (5-FU)/leucovorin (LV) has shown to improve disease-free survival (DFS) and overall survival (OS) in all age groups, along with older patients [7]. MOSAIC trial demonstrated a three-year DFS of 78.2% when oxaliplatin was added to 5-FU/LV compared to 72.9% for 5-FU/LV alone; *p* = 0.002 [8]. However, the benefit of oxaliplatin-based chemotherapy in older patients is debatable [9,10]. Moreover, the role and the benefit of perioperative chemotherapy for resectable CRLM is not yet well determined.

On the other hand, resection of CRLM shows a survival benefit. Age has been identified as a negative prognostic factor in some population-based trials that assessed the mortality of surgical resection of colorectal tumors [11]. Many physicians are reluctant to carry a curative approach in older patients, taking into consideration the associated morbidities and early mortality. Our group previously reported that hepatectomies in older patients did not have a higher 90-days mortality rate when compared to younger patients, though it carried higher risk of complications [12]. We aimed to report our center’s experience and survival analysis of older patients with CRLM who underwent curative liver resection.

## 2. Materials and Methods

We conducted a retrospective analysis of our 210 older patients who underwent hepatectomies for resectable CRLM between 2010 and 2019 after the approval of the Institutional Review Board (IRB). In the weekly multidisciplinary tumor board, CT scans were reviewed by local radiologists at diagnosis and after neoadjuvant chemotherapy, if indicated. Surgical approach and chemotherapy regimen were also discussed and determined for every patient.

Descriptive statistics were used to calculate proportions, percentages and ratios. The Charlson Comorbidity Index (CCI), a widely accepted tool for risk assessment and post-operative survival predictor, was modified and calculated with the exclusion of cancer as a weighted factor [13]. This modification was done to accurately identify the influence of comorbidities on outcomes in a cohort of patients with cancer. NLR (the absolute neutrophils count divided by the absolute lymphocytes count) was calculated based on counts collected from preoperative laboratory analyses done in our center, just before resection of liver metastases. A cut-off value of 3.0 was adopted to discriminate patients with low (NLR < 3) versus high NLR (≥3), as used in a previous retrospective trial of metastatic colorectal cancer [14].

Patients who did not experience the event of interest during data collection were censored at the date of last available follow-up. The Kaplan-Meier method was used to obtain estimates of median survival. The log-rank test was used to compare the survival curves between groups. A Cox proportional-hazards model was used to calculate hazard ratios for both univariate and multivariate analyses. Statistical tests were two-sided and a *p* value < 0.05 was considered as significant. Statistical analyses and figures were performed with R v.4.0, R Foundation for Statistical Computing (Vienna, Autria) and GraphPad Prism version 7, GraphPad Software( San Diego, CA, USA)

## 3. Results

A total of 210 patients aged 70 years and older who underwent resection of CRLM between 2010 and 2019 were identified. The median age of patients was 76 years old (range: 70–85). Mean carcinoembryonic antigen (CEA) was 82 µg/L. Some clinical and surgical characteristics are presented in Table 1.

The majority of patients had only liver metastases, and about 73% of patients had no more than two liver lesions. Localization of liver metastases and surgical approach are also summarized in Table 1. Of the 204 patients with available data, 173 (84.8%) patients had received neoadjuvant systemic chemotherapy.

**Figure 1:**
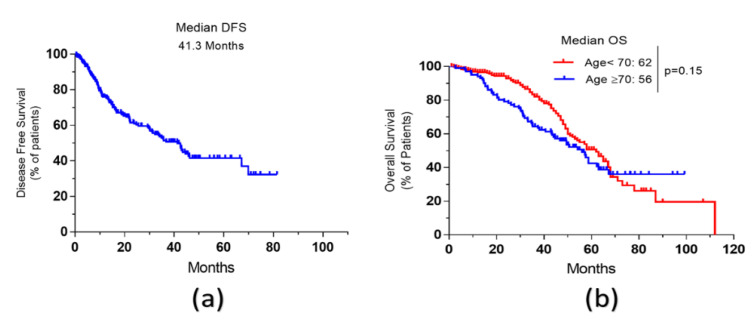
Survival Curves of older patients with resectable CRLM. (**a**): Disease-free survival (DFS). (**b**): Overall survival (OS) of older patients compared to that of younger patients.

Median disease-free survival (DFS) and overall survival (OS) of all older patients who underwent surgical resection of CRLM were 41.3 and 62.2 months, respectively. The estimated five-year survival rate was 51.5%. There was no difference in overall survival between older patients when compared to patients < 70 years of age; hazard ratio (HR) = 0.99; *p* = 0.95 (Figure 1).

**Figure 2:**
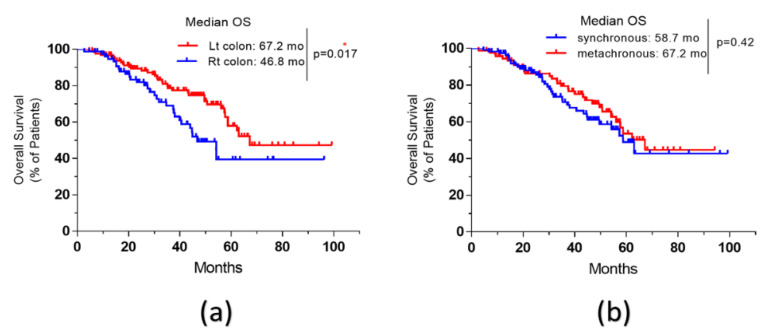
Survival curves of older patients with CRLM according to some identified factors. (**a**): overall survival (OS) of patients with right-sided primary compared to left-sided, Rt: Right; Lt: Left. (**b**): overall survival (OS) according to the timing of CRLM, synchronous versus metachronous; *: significant *p*-value.

Patients with left-sided colon cancer had a better overall survival compared to right-sided disease, with a median OS of 67.2 months compared to 46.8 months respectively, HR = 0.69; *p* = 0.017 (Figure 2a).

Patients with metachronous CRLM did not show a statistically significant improved survival compared to those with synchronous disease (67.2 vs. 58.7 months; *p* = 0.42) (Figure 2b).

**Figure 3:**
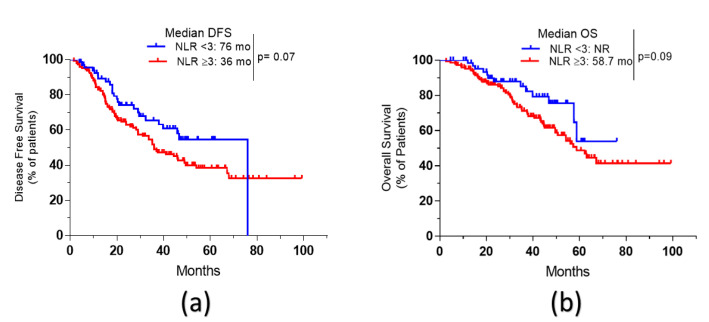
Survival curves of older patients with resectable CRLM according to NLR. (**a**): disease-free survival (DFS) of patients with NLR < 3 compared to patients with NLR ≥ 3. (**b**): overall survival (OS) of patients with NLR < 3 compared to those with NLR ≥ 3. NLR: Neutrophil/Lymphocyte Ratio.

High Neutrophil/Lymphocyte Ratio (NLR ≥ 3 versus <3) showed a trend toward a lower survival outcome. Median DFS was 36 vs. 76 months, HR was 0.68, *p* = 0.07 and median OS = 58.7 months vs. not reached, and HR was 0.63, *p* = 0.09, for high and low NLR, respectively (Figure 3).

**Figure 4:**
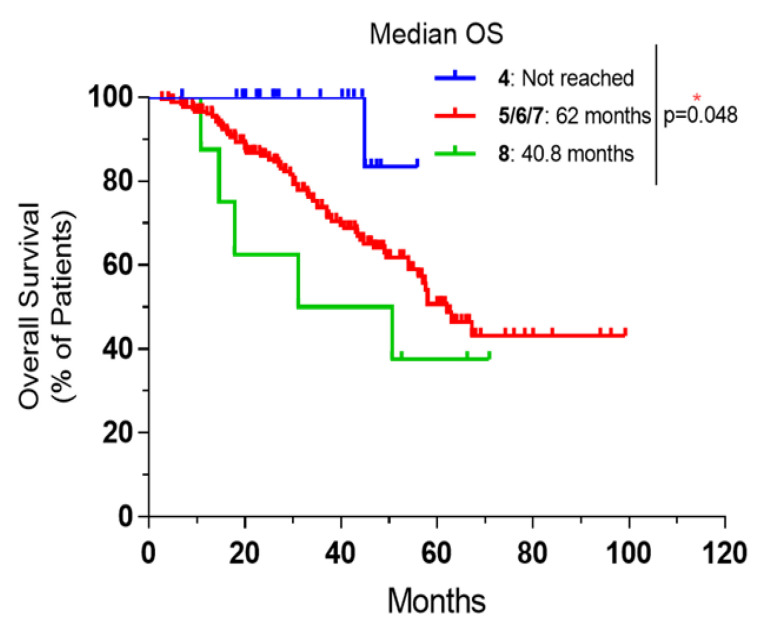
Overall survival for patients with resectable CRLM according to CCI groups. Patients were divided to three groups: CCI of 4 vs. CCI of 5, 6 and 7 vs. CCI of 8. CCI: Charlson comorbidity Score; *: significant *p*-value.

Comorbidities had shown a major impact on survival. Patients with low CCI showed the highest survival. To simplify the curve, patients with CCI of five, six and seven were combined as their survival was similar. The median OS was not reached for patients with CCI of 4, compared to 62 months for patients with CCI of five, six and seven and 40.2 months for those with a CCI of 8 (Figure 4).

**Figure 5:**
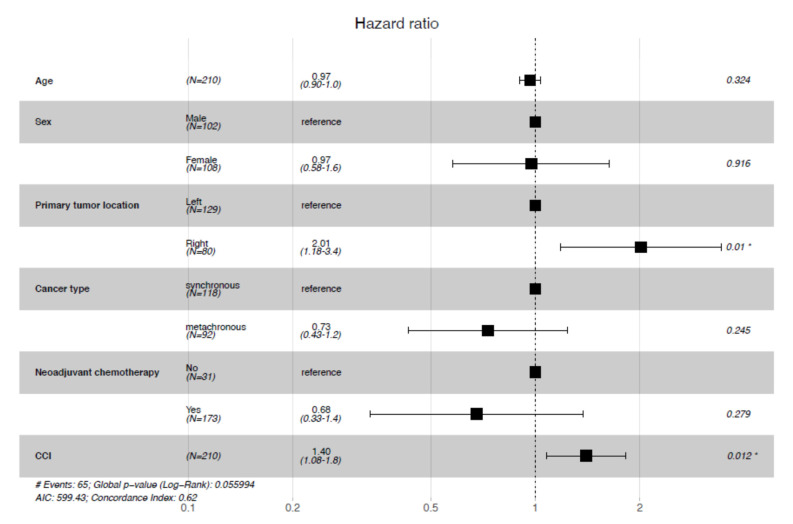
Multivariate analysis for overall survival. CCI: Charlson Comorbidity Index, *: significant *p*-value.

To control for potential confounding factors; a multivariate analysis for overall survival was performed according to certain baseline characteristics and other stratification factors. It showed that age and sex have no impact on the risk of death (*p* = 0.32 and *p* = 0.92 respectively) (Figure 5).

With respect to CCI, this analysis confirmed what was observed in a univariate setting, as a higher CCI leads to a higher risk of death (HR = 1.40, 95% Confidence Interval (CI)= [1.08–1.8].). Similarly, patients with right-sided colon cancer had a worse prognosis (HR = 2.01, 95% CI= [1.18–3.4].). No significant influence on overall survival was observed between patients who received neoadjuvant chemotherapy and those who did not, and between synchronous and metachronous metastases.

## 4. Discussion

This retrospective study showed that patients with CRLM who underwent surgical resection of the liver lesions and were aged 70 years and older had an overall survival comparable to patients less than 70 years old. It is well known that the liver is a common site of spread and metastases of tumors of the colon and high rectum, as their venous drainage is through the portal vein to the liver. Despite advances in biological targeted agents, combined with chemotherapy, survival of patients with stage IV disease remains limited, with a median of about 26–29 months [15,16]. Complete resection of liver metastases improves survival, with a median reaching up to 40 months and a five-year survival rate between 28–48% [6]. Our patients had a five-year survival rate of 51.5%, which is consistent with survival of patients enrolled in the EORTC prospective phase III trial [17]. Our study demonstrated that older patients had similar outcomes to younger counterparts, confirmed by the multivariate analysis of survival. In a systemic review, Manceau et al. demonstrated that cancer-specific survival did not decrease with age in patients undergoing surgical resection of rectal tumors [18]. In addition, age was not an independent factor for survival in older patients with limited CRLM who underwent liver resection, as shown by a propensity score matching analysis [19]. Similar survival outcomes between old and young patients were also observed in the Asian population [20]. Figueras et al. reported in a retrospective analysis that included 160 older patients, that despite higher perioperative mortality, the OS and DFS were similar to those of younger patients in recent years [21]. In a French large cohort of patients who underwent liver resection of CRLM, 20% of patients were aged more than 70 years. Although three-year survival rate was inferior to that of younger patients, 57.1% vs. 60.2%, respectively; *p* < 0.001, the group concluded that resection of CRLM in older patients can achieve reasonable survival with acceptable morbidity rates [22].

We previously reported that hepatectomies are safe in older patients and don’t cause higher postoperative mortality when compared to younger patients. However, older patients were prone to have one or more postoperative complications, especially infectious, when compared to younger patients [12]. Other reports showed that first and repeated resection of liver metastases can be safely performed in older patients [22,23,24]. Nevertheless, our study demonstrated that an increase in comorbidities leads to a decrease in survival. A systemic review and meta-analysis of 11 studies by Van Tuil et al., demonstrated that patients ≥ 70 years old had higher rates of postoperative morbidity and mortality and a lower five-year survival rate compared to younger patients. The same review included four other studies of patients ≥ 75 years old, that showed a comparable five-year survival rate to younger patients (42 vs. 32%; *p* = 0.06) [25]. There was an association observed between CCI and postoperative complications in older patients undergoing local resection of gastric neoplasms [26]. Age-adjusted Charlson comorbidity index score was an independent predictor of survival in patients with gastrointestinal tumors who underwent surgical resection in a retrospective study of 315,464 patients in China [27]. Thus, identification of comorbidities and patient assessment using screening tools and scores are to be considered rather than age. A cross-sectional study of 418 older patients with solid or hematological tumors showed that 16.7% of these patients had their initial plan of treatment changed after a comprehensive geriatric assessment (CGA) performed by a multidisciplinary team [28]. A review of evidence in 2017 concluded that CGA is feasible and can identify patients at a higher risk of adverse events such as mortality, functional decline, surgical complications and chemotherapy toxicity [29]. On the other hand, a systemic review of 178 articles, including six studies among geriatric patients undergoing major oncologic surgery, identified some predictors of postoperative complications. This review showed that no CGA predictors were identified for postoperative mortality [30]. However, the proper screening tool to predict which patients will truly benefit from a CGA is yet to be identified, as a phase II trial evaluating three different screening tools distinguished discordance in outcomes [31]. Further trials are warranted to determine the CGA effectiveness in different surgical and oncological situations.

Perioperative chemotherapy used in our center was mostly FOLFOX (Folinic Acid-Fluorouracil-oxaliplatin). Patients could receive FOLFIRI (Folinic Acid-Fluorouracil-irinotecan), also. Choice of regimen was determined according to previous oxaliplatin exposure and clinician choice. Bevacizumab was added to chemotherapy regimen according to the physician’s choice, as well as standard clinical indication and contraindications. In our study, we showed that there was no difference in survival between patients who received perioperative chemotherapy and those who did not. In the phase III EORTC 40983 prospective trial of 364 patients, perioperative chemotherapy with FOLFOX did not increase survival compared to surgery of CRLM alone [17]. On the other hand, a pooled analysis of 278 patients randomized in two phase III trials, i.e., an FFCD 9002 and an ENG trial, showed a trend to better PFS and OS with adjuvant chemotherapy compared to surgery alone after resection of liver or lung colorectal metastases [32]. It should be noted that only 20% of patients in this analysis were aged 70 years and more, whereas in the EORTC 40983 trial, older patients were not specified. Absence of postoperative chemotherapy was an independent predictor of survival in the French cohort where older patients accounted for about 20% [22]. Thus, perioperative chemotherapy is to be considered cautiously in older patients after weighing the risks and benefits.

We also observed that patients with right-sided cancer had worse prognoses. Sidedness is considered a prognostic factor, as many observational studies and clinical trials have shown. Benedix et al., and other authors, reported better overall survival in left sided colorectal tumors [33,34]. The same observation was seen in clinical trials assessing the addition of biological agents like cetuximab and panitumumab, where right-sided tumors showed a lower median overall survival across treatment arms [35,36]. These results are in contrast to a large analysis that showed no difference in survival except in stage III, where right-sided tumors carried a worse prognosis [37]. Thus, sidedness matters, especially in the choice of which biological agent to use [35,36].

In addition, a trend towards better survival with metachronous metastases was observed in our cohort. Engstrand et al. showed that the time of detection of liver metastases lacked prognostic value [38]. There was no difference in the overall survival for synchronous versus metachronous detection in their retrospective analysis. No clear pattern can be concluded regarding the significance of timing of detection in operated liver metastases [38,39,40].

There is increasing evidence that a complex interaction between tumor and host systemic inflammatory response exists and determines outcomes [41]. Elevated preoperative NLR was associated with a decrease in the disease-free survival of colorectal tumors undergoing curative resection [42,43,44]. The same results were observed in metastatic colorectal cancers receiving palliative chemotherapy, even at lower thresholds [14]. In our cohort, high NLR showed nonstatistically significant unfavorable outcomes.

Our study has several limitations, including the retrospective analysis of small number of patients, which can lead to potential bias in the conclusions drawn. As our center is a referral center for hepatic surgeries, some patients continued the management after discharge at their regional hospitals; thus we sometimes lacked detailed follow-up data, including adjuvant therapy. We also lacked proper stratification of patients, as we used mainly comorbidities. Differences in the health and function of patients can be accurately assessed using a frailty score. This was difficult to apply in this retrospective study. In addition, the results observed in our study support the evidence that older patients have similar long-term survival as younger patients with some prognostic indicators. Some of these prognostic factors were considered to have an impact on survival, based on trends observed in Kaplan-Meier curves. Preoperative NLR was evaluated retrospectively and a cut-off of 3 was selected to be consistent with other studies. The prognostic role, and its clinical implication, need to be validated in prospective trials. However, the proper patient selection method and multimodality treatments would be only established by a randomized prospective trial.

## 5. Conclusions

Resection of CRLM in older patients is feasible and demonstrated similar outcomes to younger counterparts. Chronological age should not be a contraindication to curative treatments. Therefore, older patients should be offered the same curative treatment as younger ones, with individualized management according to shared decision making. Potential comorbidities should be carefully identified, and the benefit of perioperative chemotherapy should be properly assessed to minimize perioperative complications and mortality. Consequently, multimodality treatment of CRLM in older patients should be selectively considered. Comprehensive geriatric assessment and other screening tools are to be used to properly identify these patients. Recruiting older patients in clinical trials should be encouraged.

## Figures and Tables

**Table 1:** Clinical and Surgical characteristics of the 210 patients of the cohort.

Characteristics	Number of Patients (%)
Modified Charlson Comorbidity Index	
4	25 (11.9%)
5	76 (36.2%)
6	66 (31.4%)
7	35 (16.7%)
8	8 (3.8%)
Primary Tumor Site	
Right Colon	80 (38%)
Left Colon	89 (42.3%)
Rectum	40 (19%)
Unknown	1 (0.4%)
Staging of primary tumor T stage	
T1	5 (2.4%)
T2	31 (14.8%)
T3	129 (61.4%)
T4	29 (13.8%)
Unknown	16 (7.6%)
Liver metastases only	175 (83.3%)
Liver and Lung metastases	19 (9%)
Localization of Liver Metastases	
Right Lobe	115 (54.7%)
Left Lobe	89 (42.3%)
Both	4 (2%)
Central	2 (1%)
Number of Liver Metastases	
1	116 (55.2%)
2	39 (18.6%)
3	29 (13.8%)
4	14 (6.7%)
5	6 (2.8%)
6	6 (2.8%)
Timing of liver Metastases	
Synchronous	118 (56.2%)
Metachronous	92 (43.8%)
Type of Hepatic Surgery	
Central Hepatectomy	12 (5.7%)
Right Hepatectomy	69 (32.9%)
Left Hepatectomy	41 (19.5%)
Segmentectomy not defined	79 (37.6%)
Right segmentectomy	6 (2.9%)
Left Segmentectomy	3 (1.4%)

## Data Availability

The data presented in this study are available on request from the corresponding author.
